# Small adipose-derived mesenchymal stromal cells exhibit longer telomeres and enhanced regenerative potential

**DOI:** 10.3389/fbioe.2025.1687461

**Published:** 2026-01-08

**Authors:** Uldis Berzins, Janis Baronins, Hermanis Sorokins, Saurav Goel, Andrei Shishkin, Rosita Zvirgzdina, Aija Rautmane, Danail Minchev

**Affiliations:** 1 Biochip Scientific Laboratory, Faculty of Civil and Mechanical Engineering, Mechanical and Biomedical Engineering Institute, Riga Technical University, Riga, Latvia; 2 “Turiba University” Ltd., Riga, Latvia; 3 “Stem Cell Technologies” Ltd., Riga, Latvia; 4 Faculty of Natural Sciences and Technology, Institute of Physics and Materials Science, Riga Technical University, Riga, Latvia; 5 Department of Building, Energy and Material Technology, UiT The Arctic University of Norway, Tromsø, Norway; 6 School of Engineering and Design, London South Bank University, London, United Kingdom; 7 Department of Mechanical Engineering, University of Petroleum and Energy Studies, Dehradun, India; 8 Department of Human Anatomy and Physiology, Faculty of Biology, Paisii Hilendarski University of Plovdiv, Plovdiv, Bulgaria

**Keywords:** AD–MSCs, cell rolling, cell size, BALB/c nude mice, mesenchymal stromal cells, telomere length, hypoxia

## Abstract

Younger, replicative cells with longer telomeres can enhance regenerative therapies, however, there is a lack of a standard method to assess telomere length in live cells. The present study investigated whether the relative size of human adipose tissue-derived mesenchymal stromal cells (AD–MSCs) can influence their telomere length. During early culture, a smaller-sized AD–MSC subpopulation was identified based on characteristic colony emergence. Telomere lengths in total and smaller-sized cell populations were measured. Polymerase chain reaction revealed expression of Nanog and OCT3/4 in small-sized AD–MSCs. Their safety was evaluated in immunodeficient BALB/c nude mice. Smaller AD–MSCs revealed distinct growth properties, with the cell monolayer rolling up into a large aggregate. These cells had longer telomeres (18,121.43 base pairs [bp]) than the total population (15,870.44 bp) and formed teratoma-like structures with skin-like morphology (including hair). In conclusion, AD–MSC size reliably isolates cells with longer telomeres and potential.

## 1 Introduction

Mesenchymal stromal cells (MSCs) can be isolated from various adult and fetal tissues. They possess multilineage differentiation potential and immunomodulatory properties as well as the ability to secrete a large variety of bioactive trophic factors (Murray and Péault, 2015). Historically, bone marrow is regarded as the main source of MSCs (BM–MSCs) but the 21st century discovery of adipose tissue-derived MSCs (AD–MSCs) (Zuk et al., 2001) has significantly boosted the applications of adult stromal cells in regenerative medicine (Al-Ghadban et al., 2022).

Growing evidence of inconsistent results of MSC-based therapies suggests that MSC cultures are more complex than previously thought (Olmedo-Moreno et al., 2022). They are commonly considered to be a heterogeneous mixture of cells that differ in their proliferative potential, growth properties, differentiation capacity and immunomodulatory capability (Lee et al., 2010; Russell et al., 2010; Rada et al., 2011; Whitfield et al., 2013; Zhang et al., 2022). Previous research demonstrated that human AD–MSC cultures from various donors exhibit two distinct cell subpopulations with different sizes and relative telomere lengths (RTL) (Legzdina et al., 2016).

Telomeres are short, simple, repetitive nucleotide sequences (5′–TTAGGG–3′ in humans) at the ends of eukaryotic chromosomes (Greider, 1990) that protect chromosomal deoxyribonucleic acid (DNA) and regulate cellular senescence (Shay and Wright, 2019). Each somatic cell division leads to the progressive shortening of telomeres (Harley et al., 1990) until the cell reaches its maximum proliferative lifespan and irreversible cell cycle arrest *in vitro* which is widely recognised as replicative senescence (Hayflick, 1965). MSCs intended for clinical applications are routinely propagated *in vitro* to obtain a large number of cells. This allows for controlled growth, which is necessary to ensure the safety and efficacy of cell-based therapies, but each cell doubling inevitably results in telomere shortening (Samsonraj et al., 2015; Oja et al., 2018) with a rate of approximately 100 base pairs (bp) per every two passages (Bonab et al., 2006).

It is well established that the average size of human fibroblasts increases in older cell populations (Lindrose et al., 2021). The same phenomenon can be applied to *in vitro* MSC culture. Cell enlargement, permanent growth arrest, increased activity of senescence-associated β–galactosidase (SA–β–gal) and changes in surface marker expression and differentiation potential (Cristofalo and Kritchevsky, 2008) are some of the classic characteristics of senescence and among these, the morphological transformations are most obvious and easiest to detect. Enlargement of aging MSCs through an automated image analysis system has shown that an increase in cell area can aid in identifying and sorting live, unlabelled cells by telomere length with the appearance of senescence-associated markers (Oja et al., 2018). The presence of larger, flattened AD–MSCs with irregular morphology and granular cytoplasm and increase number at later passages, has also been reported in previous studies (Legzdina et al., 2016). Research work of Legzdina et al. (2016) laid the foundation for subsequent research wherein AD–MSCs from later passages were shown to differ in size and telomere length, forming two distinct subpopulations based on their RTL. The sorting of live AD–MSCs from later passages according to their size has helped to identify a correlation between cell size and telomere length. Moreover, this label-free live cell sorting technique based on the size of AD–MSCs to separate cells according to their telomere length was patented in Latvia (patent N^o^ 15712 B1) (Berzins et al., 2023). This feasibility study was aimed at testing the possibility for sorting AD–MSCs (during isolation from adipose tissue and in early cell passages) according to their telomere length using relative cell size as the main parameter. Accordingly, minimal identity and multipotency were confirmed at the whole-population level to contextualise subsequent small-size subpopulation analyses.

## 2 Materials and methods

### 2.1 Cells isolation

Isolation of AD–MSCs from subcutaneous adipose tissue was performed in accordance with Permit No.12 issued by the Latvian Central Medical Ethics Committee as per the details described elsewhere (Bogdanova et al., 2011). For the current study, AD–MSCs were used starting from passage 0 (P0).

### 2.2 AD–MSC sourcing, viability, and batch qualification

AD–MSCs originated from the same donor and from the primary culture previously characterized (Bogdanova et al., 2011) according to International Society for Cell and Gene Therapy (ISCT) minimal criteria (Dominici et al., 2006; Viswanathan et al., 2019). After the P0, this culture was cryopreserved to establish a master cell bank. Tri-lineage (osteogenic, adipogenic, chondrogenic) differentiation was reported in a parallel study using aliquots from the same master cell bank thawed contemporaneously with the present experiments (Bērziņš et al., 2018). For each experiment, a single cryovial (“batch/lot”) from this bank was thawed and expanded to P2–P3 (Bogdanova et al., 2014). Prior to characterization, cell viability was assessed by trypan blue exclusion; only batches with viability ≥90% were advanced in the present work. Each thawed batch underwent re-qualification aligned with the ISCT minimal criteria (Dominici et al., 2006; Viswanathan et al., 2019), assessed on the total cell culture expanded from colonies arising from the very small cell fraction that yielded a homogeneous stem-cell-like population, as described previously (Bogdanova et al., 2014). Additional qualification included an immunomodulation assay based on mitogen-induced blast transformation. Only re-qualified P2–P3 batches were used in downstream assays.

### 2.3 Cells sorting and culturing

After processing adipose tissue, the stromal vascular fraction was seeded onto multiple culture flasks. Detailed conditions for culturing human MSCs have been described previously (Legzdina et al., 2016).

Human AD–MSCs used in this study have previously been characterised according to the minimal criteria for defining multipotent MSCs (Dominici et al., 2006), showing by flow cytometry the simultaneous expression of CD29, CD44, CD73, CD90, and CD105, and the absence of CD34, CD14, CD19, CD45, and HLA-DR (a major histocompatibility complex class II molecule expressed on antigen-presenting cells). It has also been demonstrated that plastic-adherent AD–MSCs from passage 6 (P6) differentiated towards adipogenic, osteogenic, and chondrogenic lineages (Bogdanova et al., 2014).

**FIGURE 1:**
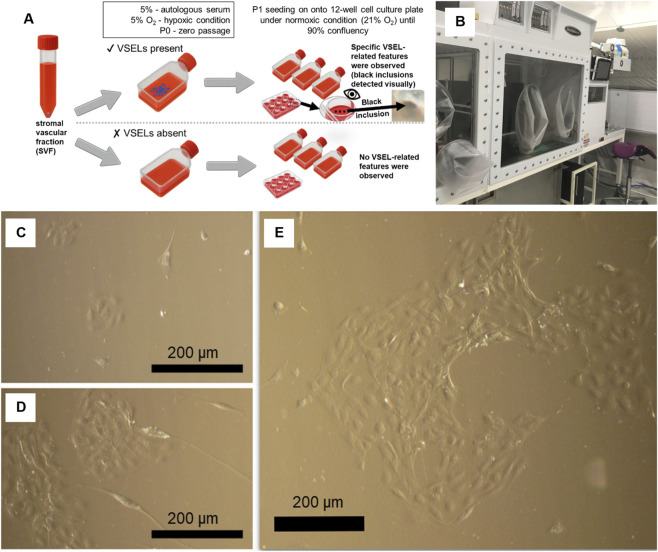
Schematic representation of cell sorting and culturing workflow. Samples marked with ✔ indicate the presence of VSELs while ✘ denoting their absence, based on morphological assessment. **(A)** The sorting procedure was carried out under hypoxic conditions within the glovebox configuration of the Xvivo System. Samples with specific VSELs related features (visually observed black inclusions) were selected for further experiments **(B)** Culturing environment within the Xvivo System. **(C)** VSELs 12 h after seeding onto the flask. **(D,E)** VSELs 24 h post-seeding, showing distinct morphological features. The presented example of the same black inclusion is fully represented in Figure 3C.

Autologous serum preparation involved blood clotting for 1 h at room temperature, followed by centrifugation at 2,000 min^-1^ for 30 min, filtration through a 0.2 μm mesh, aliquoting and storage at −20 °C. Briefly, primary AD–MSCs were expanded in 5% autologous serum, with hypoxia (5% O_2_) applied only transiently at P0—limited to a short interval immediately after isolation and plating and accounting for <20% of the P0 period. The sorting procedure was performed under hypoxia within the glovebox configuration of the Xvivo System (BioSpherix, United States of America) (Figure 1A). AD–MSCs were isolated and cultured by Stem Cell Technologies Ltd., (Latvia) using the BioSpherix Xvivo System, which provided a tightly controlled environment for cell expansion (Figure 1B).

Adipose tissue (2 mL) was cut using scissors, followed by room temperature enzymatic treatment with 0.3% pronase (EMD Millipore, United States of America) for 1 h by applying gentle rotation. The resulting suspension was centrifuged for 7 min at 1,000 min^-1^. The pellet was resuspended, passed through a 40 μm mesh filter and centrifuged for 5 min. Erythrocytes were lysed for 3 min at +37 °C using Hybri-Max erythrocyte lysis buffer (Sigma-Aldrich, United States of America). The final cell pellet was resuspended in fresh culture medium composed of basal cell culture medium DMEM/F-12 (1:1 mixture of Dulbecco’s Modified Eagle Medium–DMEM, and Ham’s F-12 Nutrient Mixture - F-12, Life Technologies, United States of America) with 10% autologous serum, 2 mM L^-1^ glutamine (Life Technologies, United States), 20 ng·ml^-1^ basic fibroblast growth factor (BD Biosciences, United States), and 100 U·ml^-1^:100 μg·ml^-1^ penicillin-streptomycin (Life Technologies, United States of America) and then seeded onto a 25 cm^2^ tissue culture flask (defined as P0). Cultures were maintained at +37 °C with 5% CO_2_ and 5% O_2_.

After 24 h, non-adherent cells were eliminated by extensive washing with phosphate-buffered saline (Life Technologies, United States of America). Remaining adherent cells continued cultivation in the medium supplemented with 10% AS for the first 10 days and 5% AS thereafter. Upon reaching 70%–80% confluence, cells were transferred to a 75 cm^2^ tissue culture flask (P1). For subsequent passages, cells were divided into three portions and seeded into three 75 cm^2^ flasks after reaching 80%–90% confluence.

Culture medium was refreshed every 72 h. Prior to adding new medium, AD–MSCs were subjected to a short rinse not exceeding 30 s with prewarmed phosphate-buffered saline.

Subsequently, the AD–MSC population was divided into two subpopulations: one without any visible small-cell colonies in the flasks (marked with ✘, Figure 1A), and another with distinct small-sized cells (Figure 1C), identified based on morphological assessment (marked with ✔, Figure 1A). The subpopulation containing Very Small Embryonic-Like Stem Cells (VSELs) exhibited characteristic features, including a small size (up to ∼10 µm), lentil-shaped morphology, clustering, rapid proliferation and transformation into larger fibroblast-like cells resembling standard MSCs.

In addition to adhesive cultures, floating cells and aggregates were detected. These included cluster formation at the medium surface, development of floating spheroids, spheroid fusion into multispheroids, and the appearance of black inclusions within spheroid clusters. Floating spheroids showed replicative youth markers, such as long telomeres (∼18 kb, confirmed by Southern blotting - SB of terminal restriction fragments), expression of pluripotency markers NANOG and OCT3/4 (detected via SB, PCR, and immunofluorescence - IF) and teratoma-like structure formation in mice, with organ-specific structures present within the teratomas-like structures.

Transformations were also observed in adhesive cultures. Small-cell expansion induced monolayer rolling, and black inclusions were found within the rolled structures. Spheroids formed on non-adhesive surfaces dissociated upon transfer to adhesive substrates, followed by the fusion of spheroids and the emergence of new aggregates exhibiting rolling behaviour after transfer.

Following the first passage (P1), both cell populations were seeded into 12-well culture plates and maintained under normoxic conditions (21% O_2_) until reaching 90% confluency. Morphological observations were continued, with VSELs revealing distinct features at 12 h (Figure 1C) and 24 h post-seeding (Figures 1D,E).

### 2.4 Telomere length assay

Telomere length was assessed in both the smaller AD–MSC population (presumed to contain VSELs) and the total AD–MSC population (presumed to have no VSELs). The analysis was conducted using the TeloTAGGG Telomere Length Assay Kit (Roche, Sigma-Aldrich, United States of America), following the manufacturer’s instructions.

### 2.5 RT-PCR for pluripotency markers

Total reverse transcriptase polymerase chain reaction (RT-PCR) was extracted from the smaller AD–MSCs and reverse transcribed into complementary deoxyribonucleic acid (cDNA). This cDNA was then used in the polymerase chain reaction (PCR) method to determine the expression of the pluripotency marker genes Nanog and OCT3/4. cDNA from the commercially available embryonic stem cell (ESC) H9 line (WA09-WiCell, United States of America) was used as a positive control. It was kindly provided as a gift from Stem Cell Technologies Ltd., (Latvia).

Ribonucleic acid (RNA) isolation from AD–MSCs employed TRI reagent (also known as TRIzol, Sigma-Aldrich, United States of America) following the manufacturer’s protocol with a slight modification. The RNA precipitation proceeded overnight at −20 °C in isopropanol containing 120 ng glycogen (Thermo Scientific, United States of America). RNA concentration and purity were evaluated using a NanoDrop® ND-1000 spectrophotometer (Thermo Scientific, United States of America). A total of 500 ng DNase-treated RNA served as input for cDNA synthesis, using the RevertAid™ First Strand cDNA Synthesis Kit (Thermo Scientific, United States of America) with oligo (dT) primer, according to the manufacturer’s protocol (Assets, 2025). 1 μL vol. of synthesized cDNA was introduced into a PCR mixture containing 2X PCR Master Mix (Thermo Scientific, United States of America) and 0.4 μM gene-specific primers (Metabion, Germany) to amplify target genes listed in the Supplementary Material section (IJSC-09-124_suppl.pdf (Legzdina et al., 2016)) of the referred article (Legzdina et al., 2016). Thermal cycling conditions included an initial denaturation at 94 °C for 3 min, followed by 30 cycles of denaturation at 94 °C for 30 s, annealing at 60 °C for 30 s and extension at 72 °C for 45 s, concluding with a final extension at 72 °C for 5 min. Amplified products were visualized through ethidium bromide staining in agarose gel electrophoresis. Densitometric analysis of gel images utilized ImageJ software (version 1.48; National Institutes of Health, United States of America).

### 2.6 Safety test of small AD–MSCs

**FIGURE 2:**
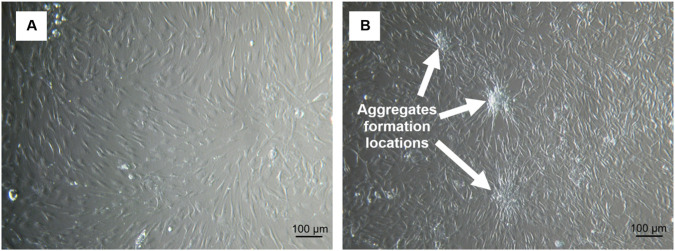
The optical microscopy images of two populations of AD–MSCs at the first passage (P1): **(A)** total cell population under normoxia; **(B)** smaller cell population identified under hypoxia (5% O_2_) through culture-based phenotypic selection from the total cell population. Arrows indicate potential aggregates formation locations.

**FIGURE 3:**
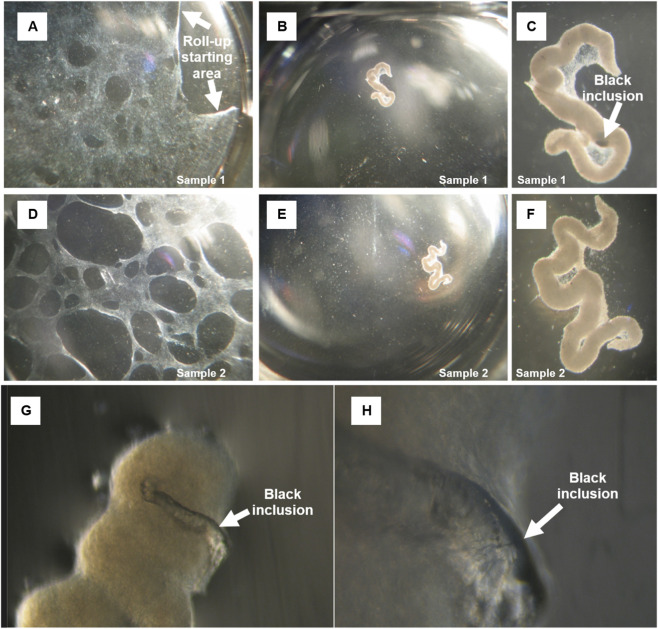
Growth properties of the small AD–MSC population demonstrated for sample 1 **(A–C)** and sample 2 **(D–F)**: **(A–D)** after forming a cell monolayer with indicated possible roll up starting area **(A)**, AD–MSCs start to roll up; **(C)** example of observable black inclusion, as demonstrated in Figure 1A; **(B,C,E,F)** 96 h after plating cells are completely rolled up, forming a large cell aggregate. **(G,H)** Floating spheroidal aggregates on the surface of the culture medium fused, after which black inclusions began to form within the spheroid clusters.

For the *in vivo* assessment of teratoma-like structure formation, subcutaneous injections were performed in two arms:

- Injection of 1·10^6^ of a human AD–MSC–containing population derived from the smaller (VSELs-like) cell population into three immunodeficient BALB/c nude mice (n = 3);
- Injection, as a suspension, of floating spheroidal aggregates formed by the same smaller (VSEL-like) cell population containing AD–MSCs into three immunodeficient BALB/c nude mice (n = 3). The aggregate formation process is shown in Figure 2B, and representative floating spheroidal aggregates are shown in Figures 3G,H. The precise cell number within aggregates and per inoculum was not determined; therefore, the dose was recorded as an “aggregate-containing inoculum” without normalization to cell number.

Sample size (n = 3 per arm) was selected for an exploratory, proof-of-concept safety assay intended to detect the occurrence of teratoma-like structures rather than to estimate their prevalence or effect size. Given the binary, high-signal endpoint and the ethical imperative to minimise animal use when working with scarce human AD–MSC preparations, three animals per condition were considered sufficient to reveal any deterministic propensity for teratoma-like formation, while acknowledging that the study is underpowered for formal statistical inference. Accordingly, the findings should be regarded as preliminary and hypothesis-generating, to be followed by adequately powered confirmatory studies if a signal is observed.

In both cases, the injection site, volume, and observation period were identical. After 29 days, the mice were euthanised, and further morphological and histological examination was done. Euthanasia was performed using cervical dislocation. The method involved allowing the mouse to grasp a grid (wire mesh). The tail is held upward at ∼30° to stabilise the body. Rapid forward and downward pressure with the thumb and forefinger at the base of the skull produces cervical dislocation, separating the brain from the spinal cord. The tail provides restraint without traction.

### 2.7 Evaluation of lineage-specific functional competence and hepatocyte-like model for HBV

Chondrogenic, osteogenic and adipogenic differentiation proceeded according to the validated protocols (Bogdanova-Jatniece et al., 2014). These assays confirmed the multipotency of the AD–MSC populations before hepatogenic induction. Evaluation of the hepatic differentiation potential of the small-sized AD–MSC subpopulation involved subjecting the cells to a directed hepatogenic differentiation protocol, as described by Taléns-Visconti et al. (2006) (Taléns-Visconti et al., 2006). It is noteworthy that this process was originally developed to compare the hepatogenic capacity of mesenchymal stem cells derived from adipose tissue and bone marrow.

The small-sized cells were cultured under defined conditions that supported endodermal lineage commitment and hepatic specification. Within several days, cells acquired a hepatocyte-like morphology (polygonal cell shape and cytoplasmic granularity) along with functional properties consistent with hepatocyte identity. The differentiated population exhibited susceptibility to hepatitis B virus (HBV) infection, a phenotype regarded as a functional hallmark of mature hepatocyte-like cells. Based on this functional competence, the hepatocyte-like culture was further developed and patented in Latvia (patent No. 14760 B) to study HBV infection dynamics *in vitro* (Zajakina et al., 2014). This model provides a reproducible and scalable system for investigating virus-host interactions under well-controlled laboratory conditions.

### 2.8 MSC characterisation and alignment with minimal criteria

All AD-MSCs batches used in this study were re-qualified at P2–P3 against the minimal criteria for multipotent MSCs. Identity was confirmed by plastic adherence and a flow-cytometric panel consistent with ISCT/IFATS guidance (CD73^+^/CD90^+^/CD105^+^; CD45^-^/CD34^-^/CD14 or CD11b^−^/CD19^-^/HLA-DR^-^ on the total adherent culture). Viability ≥90% (trypan-blue exclusion) was required prior to characterisation. Multipotency was verified by osteogenic, adipogenic and chondrogenic differentiation performed before lineage-specific experiments. Functional fitness was supported by a mitogen-induced blast-transformation assay. Only re-qualified P2–P3 batches progressed into downstream assays. The small-size subpopulation investigated for telomere length and plasticity was derived from these qualified cultures and was not intended to constitute a clinical-grade MSC product.

## 3 Results

Based on the data collected, specific VSEL-related features were observed:

- Small cells: cell size (up to ∼10 µm); lentil-shaped morphology; clustering; rapid proliferation; transformation into larger, fibroblast-like cells resembling control MSCs.
- Floating cells and aggregates were observed. Clusters emerged at the medium surface. Floating spheroids formed. Spheroids fused into multispheroids. Black inclusions were present within spheroid clusters. These phenomena were documented by visual inspection, as shown in Figure 1A. Further details are provided in Section 3.2.
- Replicative youth markers (floating spheroids): presence of long telomeres (∼18 kb, confirmed via SB); expression of pluripotency markers NANOG and OCT3/4 (detected by SB, PCR, and IF); teratoma-like formation in one of the mice; organ-specific morphology within teratoma-like structure.
- Transformation in adhesive cultures: rolling of the monolayer induced by small cell expansion; black inclusions within rolled structures; spheroid formation on non-adhesive surfaces; dissociation upon transfer to adhesive surfaces; fusion of spheroids and new aggregate formation exhibiting rolling behaviour after transfer.

### 3.1 Identification of small AD–MSCs

The total AD–MSC population was already described in Section 2. AD–MSC cells retain their fibroblast-like morphology, express typical MSC surface markers and able to differentiate into adipocytes, osteocytes and chondrocytes (Bogdanova et al., 2011; Legzdina et al., 2016; Bērziņš et al., 2018). Smaller AD–MSCs tend to form 3D aggregates and they express pluripotency markers (Bogdanova-Jatniece et al., 2014). In this work, a simple culture-based phenotypic selection allowed the identification and isolation of a distinct subpopulation of smaller cells from the overall AD–MSC population, as shown in Figures 2A,B (respectively for the total population and the smaller cells). The presence of very small cells and their specific morphological differences is clearly visible in Figures 1C–E, where VSELs at 12 and 24 h after seeding display distinct features compared to the total population.

In line with the study’s discovery scope, the ISCT/IFATS identity criteria were applied to the total adherent AD-MSC population at P2–P3 to provide context and ensure biological relevance. The subsequently isolated small-size fraction was profiled for telomere length and stemness-associated markers to interrogate heterogeneity and plasticity; a full ISCT marker panel was not the objective for this fraction, as it is not proposed here as a stand-alone clinical product.

### 3.2 Growth properties of small AD–MSCs

After the P1, a notable difference in growth properties was observed between both cell populations, as demonstrated in Figures 3A–F.

Total AD–MSC population continued to grow as expected, forming an even spindle-shaped cell monolayer. However, the small AD–MSCs started to roll up shortly after forming a monolayer (Figures 3A,D). Finally, after the plating onto a 12-well cell culture plate for 96 h, the small AD–MSC monolayer was rolled up completely, forming one large cell aggregate (Figures 3B,C,E,F). Similar cell behaviour has also been observed before but to a smaller extent (Zhu et al., 2008).

The spontaneous formation of 3D spheroid aggregates by small AD–MSCs after monolayer culture suggests reversing a more primitive, stem-like state. Such behaviour is well-documented in MSC biology, e.g., smaller, rapidly self-renewing MSC subpopulations show greater multipotency and engraftment potential than their larger and flattened counterparts (Mo et al., 2016). When cultured in 3D spheroids, MSCs become significantly smaller and less adherent, showing elevated expression of embryonic “stemness” markers (e.g., OCT4, NANOG) and enhanced secretion of trophic factors compared to 2D cultures (Kouroupis and Correa, 2021). This loss of strong substrate adhesion and shift to cell-cell interactions indicate altered adhesion molecule expression, enabling the cells to recapitulate an *in vivo*-like niche. Notably, the aggregates resemble VSEL stem cells which are rare, <6 μm cells with quiescent pluripotency that can differentiate into all three germ layers. Thus, the 3D aggregation of small AD–MSCs likely reflects a VSEL-like primitive stemness state, accompanied by changes in adhesion and signalling that poise these cells for broader differentiation pathways and regenerative potency (Kim et al., 2018).

### 3.3 Telomere length of AD–MSCs

**FIGURE 4:**
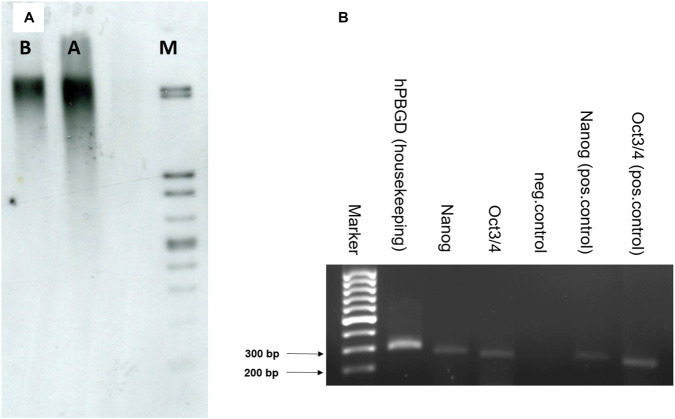
Telomere length and pluripotency marker expression. **(A)** Telomere length of AD–MSCs from the population obtained under normoxia (**(A)**, 15870.44 bp) and small cell population (**(B)**, 18121.43 bp). Marker (M). **(B)** PCR analysis of pluripotency markers Nanog and OCT3/4 in the small AD–MSCs. hPBGD – 294 bp, Nanog – 262 bp, OCT3/4–247 bp, positive control - DNA of commercial ESCs, negative control–sample without cDNA.

Telomere length was determined in both cell populations using the TeloTAGGG Telomere Length Assay Kit demonstrated in Figure 4A. The results showed that the small-cell population (lane B) exhibited significantly longer telomeres (18121.43 bp) compared to the unfiltered total AD–MSC population (lane A), which had a telomere length of 15870.44 bp. The DNA ladder in lane M served as a molecular size reference. For comparison, the telomere length of peripheral blood mononuclear cells from the same donor was previously measured at 11800.10 bp, underscoring the enhanced telomere preservation in the small-cell subset.

Figure 4B illustrates the expression of pluripotency markers assessed via RT-PCR. The small-cell AD–MSCs were positive for Nanog (262 bp) and OCT3/4 (247 bp), indicating a degree of stemness. The expression of the housekeeping gene hPBGD (294 bp) confirmed successful amplification and RNA integrity. Positive controls using commercial ESC DNA confirmed expected bands for Nanog and OCT3/4, whereas the negative control (no cDNA) showed no amplification. These results confirmed that the small-cell fraction of AD–MSCs not only possess longer telomeres but also indicates key markers associated with pluripotency.

### 3.4 RT-PCR for pluripotency markers

PCR analysis (Figure 4B) showed the expression of pluripotency markers Nanog and OCT3/4 both in ESCs, which served as a positive control, and in the small AD–MSCs. The expression of Nanog was higher, but the expression of OCT3/4 was lower in AD–MSCs when compared to the positive control.

### 3.5 Safety test

**FIGURE 5:**
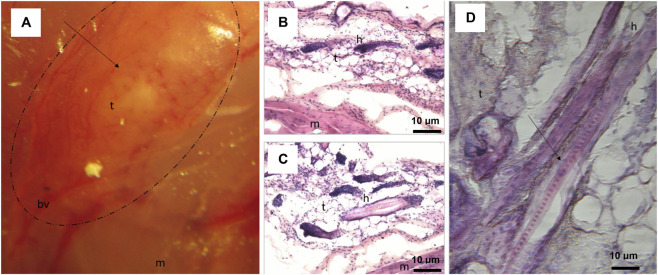
Teratoma-like structure in a BALB/c nude mouse 29 days after the small AD–MSCs subcutaneous transplantation: **(A)** subcutaneous vascularized teratoma-like structure; the arrow shows skin-like structure; **(B,C)** cross section of teratoma-like structure showing its growth in the mouse muscular tissue; hematoxylin and eosin staining; **(D)** larger magnification of the cross section; the arrow shows medulla (hair). m - muscle, h - hair, t - transplant, bv - blood vessel.

Twenty-nine days after subcutaneous transplantation of floating spheroidal aggregates derived from the smaller (VSEL-like) cell population containing AD–MSCs into BALB/c nude mice, histological analysis in one of the three animals revealed the formation of a vascularized teratoma-like structure. As shown in Figures 5A–D, the structure exhibited features resembling skin, including differentiated cell types, organized blood vessels, and the presence of hair. Cross-sectional images demonstrated the growth of the transplant within the host muscle tissue (Figures 5B,C), with hematoxylin and eosin staining highlighting tissue organization. A higher magnification view (Figure 5D) revealed hair follicle structures, including a visible medulla, further confirming differentiation within the graft. The remaining two mice did not exhibit any visually detectable changes at the injection site, and further studies are required to determine the possible underlying causes.

For the AD–MSC VSEL cohort without aggregates, teratoma-like structure formation was not observed over the 29-day observation period.

In the context of the *in vivo* safety assay, the floating spheroidal aggregate condition was considered unsafe: the assay was positive for teratoma-like structure formation (observed in 1/3 animals), indicating a potential tumorigenicity risk for this preparation. By contrast, all other tested cell preparations—including the smaller (VSEL-like) AD–MSC–containing population administered as single cells (without aggregates)—were negative in the safety assay, with no teratoma-like structures detected over the 29-day observation period.

### 3.6 Evaluation of HBV susceptibility

Small-sized AD–MSCs were subjected to hepatogenic differentiation using the protocol described by Taléns-Visconti et al. (2006) (Taléns-Visconti et al., 2006). This protocol was originally developed to assess hepatogenic potential in mesenchymal stem cells from adipose and bone marrow sources. The small-sized cell population responded readily to the differentiation cues and, within several days, developed a phenotype which was consistent with hepatocyte-like cells. Morphological hallmarks included polygonal cell shape and increased cytoplasmic granularity which are typical features associated with hepatic differentiation.

Beyond morphological transformation, the differentiated cells demonstrated a clear susceptibility to HBV infection. This functional trait is commonly regarded as a defining marker of mature hepatocyte-like cells, suggesting that the differentiation extended beyond superficial phenotypic changes and may have induced liver-specific molecular programs relevant to viral permissiveness. Based on these observations, the resulting cell model was patented as an *in vitro* system for HBV infection studies (Zajakina et al., 2014).

These findings align with previously published models of HBV infection using stem cell-derived hepatocyte-like cells. For instance (Xia et al., 2017), reported productive HBV infection in hepatocytes derived from human pluripotent stem cells, underscoring their applicability in virus–host interaction studies and Shlomai et al. (2014) developed models using hepatocyte-like cells derived from induced pluripotent and ESCs, respectively.

Notably, unlike earlier approaches, our study relied on adult stromal cells from adipose tissue which has a small-sized subpopulation of AD–MSCs that had not undergone genetic reprogramming. This distinction raises compelling questions about the latent plasticity of adult mesenchymal populations. Could certain subfractions possess a previously underappreciated capacity for endodermal differentiation? Or might their responsiveness be driven primarily by microenvironmental factors introduced during the induction process? While our current data suggest the presence of intrinsic plasticity, definitive answers will require further molecular analyses, including transcriptomic and epigenetic profiling. Nevertheless, these findings open a potential avenue for generating functionally competent hepatocyte-like cells from adult tissue sources, offering new possibilities for disease modelling and viral research platforms.

## 4 Discussion

The initial variation in the MSC population *in vitro* can be attributed to the factors such as the cells’ biological origin, the method of sample collection, the age and health of the donor, the technique used for MSC isolation and few other unknown elements (Costa et al., 2021). The presented findings align with the observation that the size of MSCs cultured *in vitro* tends to increase in later passages, accumulating larger cells over time. Studies tracking BM–MSCs have confirmed that the average cell size grows with each passage, and larger, non-dividing (senescent) cells grow in proportion (Whitfield et al., 2013). Senescent MSCs which are characterised by irreversible growth arrest exhibit diminished capabilities in differentiation, paracrine secretion and immunomodulatory function. These functional impairments render them suboptimal for regenerative applications, as they fail to adequately support tissue repair or modulate immune responses in damaged microenvironments. Some MSCs stop dividing over time. These senescent cells tend to lose much of their original function. They show poor performance in tasks like differentiation, secretion of important factors, or influencing immune activity (Turinetto et al., 2016). Due to this, they are not ideal for therapies aimed at tissue repair. Their presence can weaken the overall effectiveness of the cell preparation. To avoid this, it is important to identify and use active cells. There is also a growing trend in applying practical assessment tools to check for signs of aging in these cells before deciding on their use in treatment. These include changes in shape or expression of certain markers.

The present research demonstrates the feasibility of using the size of AD–MSC to sort cells with longer telomeres (Figure 1) and higher potential at the very beginning of the AD–MSC isolation process. This approach separates smaller cells, allowing them to obtain more potent cell cultures. Label-free cell sorting according to size can be performed using flow cytometry or dielectrophoresis, a patented method (Latvian patent No. LV 15712 B) (Berzins et al., 2023) to discriminate between cells with longer or shorter telomeres. This study is the first to demonstrate the correlation between AD–MSC size and telomere length.

While pluripotency markers Nanog and OCT3/4 are traditionally associated with ESCs, recent research has shown that they are also expressed in MSCs to varying degrees (Mikhail et al., 2008; Huang et al., 2014). In MSCs, Nanog and OCT3/4 expression is generally lower than in ESCs, but their presence indicates a certain degree of stemness and multipotency. The expression of pluripotency markers in MSCs can vary based on the tissue source and the culture conditions (Guo et al., 2019; Miceli, 2024). The results (Figure 4) showed that smaller AD–MSCs expressed both Nanog and OCT3/4, indicating a potentially higher regenerative capacity and efficiency in therapeutic applications.

Smaller AD–MSCs’ safety was tested by subcutaneous transplantation in a BALB/c nude mouse. Such a test is usually performed to observe stem cell ability to promote regeneration (Jo et al., 2021) and analyse cell toxicity, safety and efficacy (Subramani et al., 2022). The present research was aimed to test the safety of floating spheroidal aggregates (Figures 3G,H) that formed of AD–MSCs and the analysis revealed a teratoma-like structure with skin-like morphology, akin to a ‘skin under skin’ phenomenon (Figure 5). Different types of cells were observed resembling skin structure, including hair. Whether the hair originated from human or mouse stem cells that could migrate through the established teratoma blood vessels remained unclear. They could also be hybrid formation of mouse and human origin. Teratoma formation tests are typically used in embryonic or induced pluripotent stem cell studies (Zhang et al., 2008; Hentze et al., 2009; Prokhorova et al., 2009; Gropp et al., 2012). Adult stem cells do not form teratomas, but they stimulate regenerative processes (Cable et al., 2020). No teratomas were observed with small AD–MSCs that exhibited no aggregates formation (Figure 2B). This supports an acceptable safety profile. Their responsiveness to microenvironmental cues indicates a broad, lineage-restricted differentiation capacity. Skin-like structures can be generated. VSELs did not form teratoma-like structures under the conditions examined. This further supports their safety. In contrast, non-adherent aggregates that transition into free-floating spheres enter an altered and unstable state. Phenotypic drift is apparent. Such cultures are unacceptable for therapeutic use. They should be excluded by quality-control criteria unless specifically controlled and justified.

Smaller AD–MSCs were found to contain longer telomeres in comparison to the broader cell population (Figure 4). This variation in telomere length suggests that physical size may correlate with cellular potency. Using size as a sorting parameter allows for the separation of cells that are more likely to retain regenerative properties. This approach is simple in design and adaptable to multiple areas, such as tissue repair, therapeutic product development, compound screening and cosmetic formulation (Vasanthan et al., 2020; Yin et al., 2020). Applications involving such cells may contribute to strategies aimed at reducing age-associated degeneration.

The present study demonstrates that a seemingly homogeneous population of human AD–MSCs is in fact heterogeneous, comprising subpopulations that differ in telomere length. In previous study, using Flow-FISH, two subpopulations were identified: smaller cells with shorter telomeres and larger cells with longer telomeres (after cell processing) (Legzdina et al., 2016). Hypothetically, the initial data suggest that VSEL-like cells correspond to the fraction with relatively longer telomeres. Such a possibility of discrimination is maintained across several passages. The ability to isolate these cells from a live, unlabelled culture has been patented (Berzins et al., 2023).

Differentiation of small-sized AD–MSCs into hepatocyte-like cells resulted in a population exhibiting classical hepatic features and the capacity to support HBV infection. This outcome aligned with earlier reports based on induced pluripotent and ESCs-derived hepatocyte-like models. However, a key distinction lies in using unmodified adult stromal cells as the starting material. The resulting cells were susceptible to viral entry. They included in a larger framework aimed at improving preclinical models for HBV-related research, including studies of viral pathogenesis and early-stage vaccine assessment (Jahnavi et al., 2022).

Under defined hepatogenic culture conditions, adapted from the Taléns-Visconti protocol (Taléns-Visconti et al., 2006), small AD–MSCs acquired a transitional phenotype termed “H2” cells. This subset demonstrated the ability to replicate HBV, as validated through several assays such as real-time RT-PCR, enzyme-linked immunosorbent assay (ELISA) for surface and envelope antigens (HBs, HBe), and electron microscopy confirming the presence of viral particles. Neutralization assays employing anti-preS1 monoclonal antibodies (MA18-7) confirmed infection specificity by blocking HBV entry in undifferentiated and differentiated cell types. Evaluation of HBV messenger RNA (mRNA) further confirmed active infection and replication within the cultures.

These findings support the relevance of H2 cells in modelling early HBV infection, particularly in experimental settings where standard cell lines provide low virus output or inconsistent results. The modified H2 line, optimised for commercial applications under the identifier 4bHHI, demonstrated improved proliferation and viral susceptibility. This system offers a practical and scalable solution, providing an ethically sound alternative to models based on embryonic or animal-derived cells. It also holds utility in screening compounds and vaccine formulations.

Generating HBV-sensitive hepatocyte-like cells from adult tissue without genetic manipulation prompts further biological inquiry. The potential role of latent plasticity within small AD–MSCs or microenvironmental influences such as the exposure to epidermal growth factor (EGF) and basic fibroblast growth factor (bFGF) remains unclear. Ongoing and future efforts may focus on transcriptomic and functional analyses to delineate the mechanisms involved. Small-sized AD–MSCs show promise for use in translational applications, with relevance extending beyond regenerative strategies to include experimental systems for studying viral infections and evaluating therapeutic compounds. Their consistent response in HBV-related assay supports their value in basic investigations and practical biomedical development.

Several studies provide compelling evidence that VSELs in adult humans may become activated in response to psychological stress. A key study published in Neurochemical Research demonstrated that social isolation stress (SIS) in mice led to a significant increase in VSEL levels in bone marrow, peripheral blood and the hippocampus (Haj-Mirzaian et al., 2020). This mobilization was associated with improved behavioural test outcomes and reduced neuroinflammation, suggesting a potential protective role for VSELs under psychological stress. Additionally, other reports indicate that VSELs can be mobilised in adult organisms following stroke or pharmacological stimulation and may reside in the adult brain as a dormant, pluripotent reserve. Another study showed correlations between VSEL markers (e.g., CD133^+^) and clinical symptoms in patients at ultra-high risk of psychosis and in first-episode psychosis. This supports the hypothesis that psychological or neuropsychiatric states can influence the activity or presence of VSELs. These findings collectively introduce a novel paradigm in which adult VSELs not only respond to somatic injury but also to mental or emotional stressors, potentially contributing to neuroprotection, regeneration or homeostatic regulation in the central nervous system.

One of the future studies could be focused on combining chitosan-based hydrogels with AD–MSCs. This approach may theoretically promote faster regeneration of damaged skin; however, further studies are required to confirm the efficacy of such an approach (Liu et al., 2023; Wu et al., 2024; Ezati et al., 2025). A chitosan hydrogel functionalised with an insulin-like growth factor 1 C-terminal (IGF-1C) peptide was shown to provide an artificial microenvironment that significantly enhances the survival and functionality of AD–MSCs (Feng et al., 2016). Research indicates that chitosan can support stem cell function by maintaining a favourable niche for a prolonged period, enhancing paracrine factor secretion, and promoting angiogenesis while reducing inflammation (Rafie and Meshkini, 2021; Kim et al., 2023; Liu et al., 2023; Zhang et al., 2024). These properties highlight the potential of chitosan as a supportive scaffold in regenerative medicine for its application in skin repair and stem cell-based therapies (Feng et al., 2016).

### 4.1 Dormant state of AD–MSC aggregates and activation upon migration

Findings in the present article indicate that spontaneously formed floating aggregates of AD–MSCs (see Figures 3G,H) represent a primitive, low-activity cell state. Previous observations for the same AD–MSC (by Bogdanova-Jatniece et al., 2014) show that the cells were dormant-like, alkaline phosphatase (AP)-negative, but expressed the pluripotency marker NANOG, consistent with a less differentiated subpopulation. When transferred onto adherent plastic, AD–MSCs migrated out of the aggregates, became AP-positive, and adopted a more active phenotype. The increase in AP activity suggests that these migrating cells may have regained differentiation capacity. Consistently, differentiation assays of AD–MSC aggregates into chondrogenic, osteogenic, and adipogenic lineages were unsuccessful, most likely reflecting the low AP activity within the aggregates. Taken together, these results support a model in which AD–MSCs within aggregates remain in a primitive and quiescent state. At the same time, the migratory cells released from these structures acquire properties indicative of higher differentiation potential (Bogdanova-Jatniece et al., 2014).

### 4.2 Oxygen shift as a stress trigger for aggregate formation

Observations in the present article suggest that the spontaneous formation of AD–MSCs or VSELs aggregates (Figures 3G,H) and roll up (Figures 3B,C,E,F) after transfer from hypoxia to normoxia (21% O_2_) may be interpreted as a stress response to sudden oxygen upshift (Estrada et al., 2012). Stem cells are physiologically adapted to low-oxygen (relatively hypoxic) niches *in vivo*, typically ranging between 2% and 7% O_2_ in bone marrow and adipose tissue, while atmospheric oxygen tension is supra-physiological for these cells (Mohyeldin et al., 2010). The sudden rise in oxygen availability can act as a strong environmental stressor for the cells.

Previous studies have demonstrated that culturing MSCs under normoxic conditions leads to increased production of reactive oxygen species (ROS). High ROS concentration cause accumulation of DNA damage, and reduced genomic stability compared with hypoxic conditions (Estrada et al., 2012). Long-term exposure to 21% O_2_ accelerates cellular senescence, while hypoxic culture delays this process and preserves stemness (Fehrer et al., 2007). The hypoxia-to-normoxia transition is also associated with downregulation of hypoxia-inducible factor HIF-1α and a metabolic shift toward oxidative phosphorylation, both of which can decrease proliferation and enhance apoptotic susceptibility (Mohyeldin et al., 2010).

In this context, the formation of floating aggregates by AD–MSCs or VSELs or roll up following oxygen transition may represent a protective mechanism, allowing cells to cluster together and enter a transient dormant state. Upon reattachment to plastic, these aggregates give rise to migrating cells that re-activate, display AP activity, and demonstrate signs of higher differentiation potential, as observed in detail in previous work (Bogdanova-Jatniece et al., 2014). Above discussed interpretation is consistent with our results, where AP-negative aggregates contrasted with AP-positive migrating cells, suggesting that oxygen-induced stress may underlie both the dormancy and subsequent reactivation of these stem cell populations.

### 4.3 Quiescent-like spheroids, potency on adhesion

Observation in the present work indicate that under floating spheroid conditions (Figures 3G,H), cells adopt a low-activity, likely quiescent (G0) state characterized by absent differentiation and minimal expression of canonical pluripotency/differentiation markers; transfer to an adhesive substrate induces migration from aggregates and restoration of functional activity, including alkaline phosphatase positivity, consistent with exit from quiescence and a “return of potency.” *In vivo*, a fraction derived from floating spheres forms teratoma-like formation in immunodeficient mice, whereas adherent AD–MSC controls do not; given the generally non-tumorigenic behaviour reported for MSCs and related populations and safety data summarized in the World Journal of Stem Cells review (López-Iglesias et al., 2011), this contrast points either to a potent component within the sphere fraction or to possible contamination, warranting rigorous verification of identity, pluripotency, and genome stability. Overall, the interpretation aligns with established concepts in AD–MSCs biology and with ISCT and International Federation for Adipose Therapeutics and Science (IFATS) minimal criteria for AD–MSC identity and functional readouts, whereby tri-lineage differentiation is typically demonstrated following cellular activation/expansion rather than under strict G0 conditions (Bourin et al., 2013).

### 4.4 Black inclusions signal hypoxic cores in MSCs spheroids and roll up aggregates

Adipose perivascular microenvironments are hypoxic and harbour MSCs, which can be isolated and expanded under 1%–5% O_2_ (Crisan et al., 2008; Estrada et al., 2012; Antebi et al., 2018; Lupatov and Yarygin, 2022). In such conditions, MSCs remain small, highly clonogenic, and readily form adherent aggregates and free-floating spheroids (Rosová et al., 2008; Basciano et al., 2011).

Diffusion constraints in 3D cultures establish a stable radial oxygen gradient once spheroids reach approximately 200–300 μm in diameter, with HIF-1α stabilised in the hypoxic core and degraded towards the rim; larger constructs (>500–900 μm) commonly develop necrotic centres (Grimes et al., 2016; Huang et al., 2017; Schmitz et al., 2021). Similar necrotic centres may also be evident in Roll-Up aggregates, as demonstrated in Figures 3C,G,H.

Exposure of peripheral layers to atmospheric oxygen (21% O_2_) intensifies oxidative stress, further accentuating the core–rim contrast (Mas-Bargues et al., 2019).

Hypoxic cores act as organiser-like foci by secreting chemotactic and trophic cues, notably SDF-1/CXCL12 and VEGF, alongside hepatocyte growth factor (HGF), epidermal growth factor (EGF) and basic fibroblast growth factor (bFGF) (Liu et al., 2013; Korbecki et al., 2021; Yang et al., 2022). Hypoxia-expanded MSCs exhibit increased motility towards these gradients, associated with upregulated chemokine receptors (e.g., CXCR4/CX3CR1/c-Met) and enhanced Ras homolog family member A (RhoA) activation (Hung et al., 2007; Das et al., 2010; Silvestro et al., 2022). Directed migration under hypoxic preconditioning increases, typically by ∼1.3–1.6× rather than 2–3×; for example, 24 h at 1% O_2_ raised SDF-1α–driven chemotaxis from ∼140 to ∼220 cells per 100× field in a Boyden-chamber assay, and the increase was abrogated by anti-CXCR4 (Hung et al., 2007). Comparable hypoxia-enhanced motility has been observed in scratch/transwell assays and linked to HIF-1α–dependent upregulation of chemokine signalling (Rosová et al., 2008).

Beyond recruitment, hypoxia modulates cell state (Mohyeldin et al., 2010). HIF-dependent programs can re-induce pluripotency-associated factors (OCT4, SOX2, NANOG) and promote dedifferentiation, thereby increasing developmental plasticity (Covello et al., 2006; Fotia et al., 2015).

During embryogenesis, oxygen tension is low (∼1%–5% O_2_), with morphogenesis dependent on intact HIF signalling. In 129/SvJ Dnd1^Ter/+^ mice, acute foetal hypoxia (10% O_2_ for 12 h at E13.8–E14.3) increased bilateral testicular teratoma frequency from 3.3% to 64% and preserved OCT4/SOX2/NANOG expression as well as TGF-β family morphogen (Nodal) activity (Bustamante-Marin and Capel, 2023). *In vitro*, human pluripotent stem cells spheroids self-organise into skin organoids with dermal and stratified epidermal components and form hair follicles, demonstrating organiser-like behaviour in pluripotent aggregate systems (Dunwoodie, 2009; Imanirad and Dzierzak, 2013; Lee et al., 2020; Bustamante-Marin and Capel, 2023).

An orthogonal axis is provided by telomere biology. In hypoxic niches, quiescent stem cells maintain longer telomeres and defer replicative senescence, thereby prolonging the interval during which organiser behaviour and high potency are supported (Nishi et al., 2004; Lupatov and Yarygin, 2022). Small clonogenic MSCs are likewise sustained under low O_2_ and can be efficiently expanded from adipose tissue (Estrada et al., 2012; Antebi et al., 2018; Chen et al., 2018).

These features also offer practical read-outs (Godet et al., 2022). Hypoxic/necrotic cores present as optically dark inclusions; HIF-1α or pimonidazole staining highlights hypoxic regions, and optical coherence tomography (OCT) detects central attenuation and quantifies core formation (Huang et al., 2017; Godet et al., 2022).

The integrated model yields testable predictions. Maintaining spheroids below the hypoxic threshold (∼200 μm) should attenuate HIF-1α stabilisation and diminish organiser activity, whereas controlled enlargement should intensify paracrine signalling and chemotaxis (Grimes et al., 2016; Schmitz et al., 2021; Ohori-Morita et al., 2025).

Pharmacological blockade of SDF-1/CXCR4 (e.g., plerixafor AMD3100 - selective CXCR4 antagonist) diminishes chemotactic ingress and directional persistence in 3D gradient assays that mimic spheroid environments; on a collagen-embedded microfluidic platform, AMD3100 abolished MSCs directionality up an SDF-1α gradient, and CXCR4/CXCR7 blockade reduces hypoxia-primed MSCs chemotaxis/homing *in vitro* and *in vivo* (Liu et al., 2012; Park et al., 2017).

Perturbation of RhoA/ROCK (Rho-associated coiled-coil containing protein kinase) signalling shows a clear dimensional context. In 3D matrices, inhibition with C3 transferase or Y-27632 diminishes directional persistence while leaving cell speed essentially unchanged (Hwang et al., 2014). In 2D or Transwell assays (porous membrane insert system in migration/chemokine axes, Boyden-type), RhoA/ROCK inhibition may instead increase chemotactic migration (Jaganathan et al., 2007). Under hypoxic preconditioning, receptor upregulation (CXCR4/CX3CR1/c-Met) and RhoA activity track with enhanced directed migration, though RhoA responses are protocol-dependent: hypoxia increases CXCR4/CX3CR1 and c-Met (Hung et al., 2007; Chacko et al., 2010), with reports of increased RhoA-guanosine triphosphate (GTP) accompanying greater directionality (Vertelov et al., 2013) and, in other settings, HIF-1α–linked reductions in RhoA-GTP and migration (Raheja et al., 2011). Longitudinal oxygen mapping, OCT, and cytokine profiling provide orthogonal validation of niche identity and function (Dmitriev et al., 2013; Raza et al., 2017; El-Sadek et al., 2021; Yang et al., 2022).

Collectively, convergent evidence from perivascular niche biology, three-dimensional oxygen-diffusion constraints, and HIF-mediated maintenance of stemness supports a coherent organiser-like framework that accounts for aggregate-driven recruitment and hypoxia-modulated cell-state plasticity in adipose-derived MSCs systems (Covello et al., 2006; Crisan et al., 2008; Grimes et al., 2016).

Robust skin/“dermatomal-like” outcomes via self-organising aggregates are firmly established in pluripotent contexts (human pluripotent stem cells skin organoids with appendages). In contrast, in adipose-derived MSCs systems, the literature predominantly reports keratinocyte-like differentiation under defined cues, though the findings are less definitive (Chavez-Munoz et al., 2013; Lee et al., 2020).

### 4.5 Deep quiescence preserves embryonic-like stem cell potency

Findings in the present work and literature support the hypothesis that a population of “true” stem cells with embryonic-like properties persists throughout life. Extreme quiescence is proposed as the principal mechanism that preserves potency while maintaining organism-wide structural integrity and healthy ageing (Tümpel and Rudolph, 2019).

This view aligns with convergent evidence. Quiescence can broaden the developmental potential of naïve ESCs, allowing for both embryonic and extraembryonic fates (Khoa et al., 2024), Adult tissue stem cells are long-lived, niche-dependent, and progressively remodelled with age, and rejuvenation is achievable by targeting niche cues (Brunet et al., 2023).

In the nervous system, *in vivo* imaging shows that adult neurogenesis persists. The rate slows with age, consistent with shrinkage and deepening quiescence of the stem-cell pool rather than its disappearance (Wu et al., 2023).

Mechanistically, culture handling was designed to respect the natural microenvironment of quiescent cells, emphasising rapid isolation and brief, early “physioxic/hypoxic” support. In the present study, hypoxia was applied only transiently at P0, confined to a short interval immediately after isolation and plating (<20% of the P0 period). Subsequent culture proceeded under normoxia. This approach provided physiological support without chronic oxygen reduction.

Two lines of evidence justify this design. Engineered niches that preserve low-oxygen, perivascular signalling maintain long-term hematopoietic stem cells (HSCs) without cytokine cocktails (Donnelly et al., 2024). Physioxia selectively enriches *bona fide* HSCs over progenitors (Igarashi et al., 2023). Direct measurements also show that incubator set-points often misrepresent true pericellular O_2_. Dense cultures can drift toward hypoxia or anoxia, underscoring the importance of carefully timed oxygen control immediately after isolation (Rogers et al., 2024).

Short hypoxic preconditioning can enhance MSC survival and pro-angiogenic programmes while reducing senescence signatures. These effects support a brief, early exposure rather than chronic hypoxia (Sohi et al., 2024).

The observations—including rare, highly plastic structures—are consistent with an embryonic-like, quiescent reservoir that niche-level perturbations can unmask. Rigorous, context-matched validation is required, with emphasis on niche reconstruction, oxygen micro-monitoring, and longitudinal assays of potency under defined physioxia.

### 4.6 Removing floating spheroidal aggregates safeguards AD–MSC product safety

Present research indicates that hypoxia, when used as a research-stage stressor, unmasks a floating aggregate (spheroid) fraction with distinct behaviour, as demonstrated in Figures 3G,H. This fraction is associated with teratoma-like (dermoid-type) structures *in vivo*. By contrast, the clinically relevant, plastic-adherent AD–MSC population that meets ISCT/IFATS criteria does not exhibit such a signal (Dominici et al., 2006; Bourin et al., 2013). This fraction-specific contrast is consistent with the accepted identity framework for AD–MSCs and supports restricting clinically intended material to the adherent phenotype.

The present work interrogated hypoxia because adipose tissue resides in a comparatively low-oxygen niche. Numerous studies show that reduced oxygen tension (≈3–5% O_2_) can modulate MSC proliferation, senescence, and paracrine activity through HIF-dependent pathways (Rosová et al., 2008; Tsai et al., 2011; Kwon et al., 2017; Schmitz et al., 2021; Moniz et al., 2022). Such conditions are valuable for discovery, but hypoxia and/or spheroidal growth can also induce quiescence and intrinsic spheroid hypoxia. These effects alter differentiation readiness and marker expression, mirroring the features observed in the floating fraction. In the present work, hypoxia served as a deliberate research tool to chart the boundary between desirable reparative potency and undesirable instability. Recent findings highlight limitations in reproducibility and safety; thus, the procedure is not currently included in clinical manufacturing (Rosová et al., 2008; Tsai et al., 2011; Kwon et al., 2017; Schmitz et al., 2021; Moniz et al., 2022).

From a translational standpoint, any *in vivo* teratoma or teratoma-like read-out is clinically unacceptable. Contemporary guidance from the International Society for Stem Cell Research (ISSCR, United States of America), together with the U.S. Food and Drug Administration (FDA, United States of America), Center for Biologics Evaluation and Research (CBER, United States of America) and the European Medicines Agency (EMA, European Union) frameworks and the Health and Environmental Sciences Institute (HESI, United States of America)/ISCT consensus papers, mandates systematic tumorigenicity risk assessment and robust control of residual undifferentiated states (Committee for Advanced Therapies CAT, 2011; U.S. Department of Health and Human Services, Food and Drug Administration, Center for Biologics Evaluation and Research, 2013; Sato et al., 2019; Lovell-Badge et al., 2021). Accordingly, the clinical process is fixed to normoxia and adherent expansion, and floating/aggregate material is categorically excluded from specification. Oxygen tension is treated as a critical process parameter, and layered quality control—ISCT/IFATS identity, absence of pluripotency features, and genomic stability—serves as release criteria.

Histologically, the classical teratoma assay requires demonstration of derivatives from all three germ layers within a single lesion. Skin and adnexal structures are predominantly ectodermal and, considered in isolation, do not fulfil this definition (Gropp et al., 2012; Montilla-Rojo et al., 2023; Ahmed and Lotfollahzadeh, 2025). Nevertheless, teratoma-like findings constitute a material tumorigenicity signal that justifies exclusion of the aggregate fraction and reinforces stringent quality controls in clinical workflows. In rodents, adherent AD–MSCs expanded under normoxia did not form tumours, whereas the hypoxia-derived floating fraction did. In an independent veterinary context, adherent adipose-derived preparations administered to dogs at high dose with multi-year follow-up produced no tumour formation or related adverse events. Taken together, these observations support a fraction-specific risk model and the practical decision to prevent spheroid formation during manufacturing (Bērziņš et al., 2018).

These findings support a model in which cell-state plasticity—the “window” of potency—is oxygen-sensitive: excessive potency risks teratoma-like outcomes, whereas excessive stress or forced differentiation risks functional attrition or apoptosis. Practically, this argues for a conservative, clinically oriented envelope (normoxia; adherence) while retaining hypoxia only as a discovery stress-test to define “do-not-cross” boundaries and sharpen quality controls, including identity, pluripotency surveillance, genomic stability, and *in vivo* biodistribution/tumorigenicity testing in appropriately powered studies (Bedel et al., 2017).

### 4.7 Validation of small stem cell findings

Research over the past two decades has reported the presence of rare, very small stem cells in adult tissues that exhibit features of pluripotency or broad developmental potential (Parker, 2014; Simerman et al., 2014). These include VSELs, initially described in bone marrow and other organs, and related populations such as multilineage-differentiating stress-enduring (Muse) cells isolated from MSC (Simerman et al., 2014). These cells are typically much smaller than ordinary stromal or blood cells (often on the order of 3–6 μm in diameter), reside in quiescent states in adult tissues, and express ESC markers like OCT4 and Nanog while lacking hematopoietic lineage markers. Telomere biology is of particular interest: unlike typical adult somatic cells, some of these small stem cells have exhibited telomerase activity or relatively long telomeres consistent with primitive or stem-like status. If confirmed, such cells could have significant implications for regenerative medicine and aging, by serving as a reservoir of pluripotent/multipotent cells in adult organs (Cancedda and Mastrogiacomo, 2024).

However, this field has remained controversial. Some groups have had difficulty reproducing the isolation of VSELs, leading to debates about their existence (Parker, 2014). The characteristics and nomenclature of these cells also vary across studies, raising concerns that some reports might represent artifacts or misidentified cell fragments.

As a result, a comprehensive table has been compiled in Supplementary Table S1 - Article_Analysis (list of used abbreviations demonstrated in the sheet Supplementary Table S1 - List of used abbreviations), summarizing data from 98 literature sources by systematically compiling the following:

- Study ID
- Donor tissue source (from 1 to 4, as found in different articles)
- Was VSEL presence tested?
- Was VSEL detected?
- Total cell isolation method
- VSEL cell isolation method
- Observed VSEL size or size range (μm)
- Number of base pairs in VSEL population (bp)
- Number of total base pairs in cell population (bp)
- Marker List
- Functional Test
- Donor species
- Confirmation method
- Do total colonies “roll up” on the plate?
- Do VSEL colonies “roll up” on the plate?
- Do colonies form “spheroids” or lumps that float?
- Do VSEL colonies form “spheroids” or lumps that float?
- Teratoma formation in mice?
- Conclusion from teratoma formation test in mice?
- Pluripotency factors (NANOG, OCT4) and telomere length studied?
- Conclusions from pluripotency factors tests.
- What cell markers/parameters were characterized?
- Conclusions from markers/parameters characterization
- Aim of the study
- Main conclusions

The review of 98 articles (see Supplementary Table S1 - Article_Analysis) comprises a total of 98 studies, each classified by the primary donor source used for cell derivation, as shown in the Table 1. The most prevalent donor source is human umbilical cord blood, represented in 40 studies. Animal bone marrow follows, with 25 studies. Human peripheral blood was used in 11 studies, while other animal-derived sources such as ovary (4 studies), uterus (4), testis (4), and pancreas (1) collectively account for a smaller proportion. Human-derived source cases like bone marrow from cadavers or patients (3 studies), bone marrow (non-specified, 3), testis (2), Wharton’s jelly/placenta (1), and heart (1) were relatively rarely reported in the literature.

**TABLE 1:** Study grouping by donor origin (human/animal) and tissue source with corresponding study IDs (Supplementary Table S1 - Article_Analysis).

Main donor source	Study IDs^reference^	Number of studies
Human - Umbilical cord blood	2^(Mahmoudi et al., 2021)^, 4^(Chang et al., 2014)^, 7^(Domingues et al., 2022)^, 8^(Gounari et al., 2019)^, 9^(Tavakoli et al., 2017)^, 10^(Finkel et al., 2023)^, 15^(Kuruca et al., 2019)^, 18^(Akhlaghpour et al., 2020)^, 19^(Zarei−Behjani et al., 2019)^, 22^(Santourlidis et al., 2011)^, 29^(Trompeter et al., 2013)^, 30^(Vinukonda et al., 2019)^, 34^(Halasa et al., 2008)^, 37^(Schaap−Oziemlak et al., 2010)^, 39^(Bhartiya et al., 2012b)^, 40^(Danova−Alt et al., 2012)^, 42^(Biazar and Heidari Keshel, 2015)^, 46^(Bujko et al., 2023a)^, 48^(Ghanjati and Santourlidis, 2016)^, 51^(Shafiee et al., 2011)^, 53^(Schorn et al., 2022)^, 54^(Zuba−Surma et al., 2010)^, 55^(Sensken et al., 2007)^, 56^(Sahmani et al., 2018)^, 57^(Purohit et al., 2021)^, 58^(Greschat et al., 2008)^, 61^(Hedayati et al., 2018)^, 63^(Bahrami et al., 2016)^, 64^(Li et al., 2017)^, 65^(Iwaniuk et al., 2011)^, 68^(Jamshidi−Adegani et al., 2014)^, 70^(Jeltsch et al., 2011)^, 71^(Waclawczyk et al., 2010)^, 75^(Kucia et al., 2007)^, 76^(Langroudi et al., 2013)^, 84^(El Baz et al., 2019)^, 86^(Wernet et al., 2010)^, 89^(Schira et al., 2015)^, 93^(Ruseva et al., 2024)^, 97^(Bujko et al., 2024)^	40
Animal - Bone marrow	3^(Shin et al., 2012)^, 6^(Curtis et al., 2012)^, 12^(Sun et al., 2020)^, 13^(Miyanishi et al., 2013)^, 21^(Maj et al., 2015)^, 24^(Wu et al., 2012)^, 25^(Mierzejewska et al., 2013)^, 32^(Grymula et al., 2014a)^, 38^(Labedz−Maslowska et al., 2016)^, 43^(Abouzaripour et al., 2015)^, 45^(Shin et al., 2010)^, 47^(Golipoor et al., 2016)^, 50^(Shaikh et al., 2016)^, 67^(Grymula et al., 2014b)^, 69^(Zuba−Surma et al., 2011)^, 72^(Chen et al., 2015)^, 73^(Kassmer et al., 2013)^, 80^(Dawn et al., 2008)^, 81^(Grau−Monge et al., 2017)^, 87^(Sovalat et al., 2011)^, 90^(Rahnemai−Azar et al., 2011)^, 92^(Roger et al., 2012)^, 95^(Chumak et al., 2023)^, 96^(Bujko et al., 2023b)^	24
Human - Peripheral blood	5^(Wojakowski et al., 2009)^, 14^(Drukała et al., 2012)^, 23^(Zbucka−Kretowska et al., 2016)^, 27^(Sovalat et al., 2016)^, 35^(Havens et al., 2013)^, 44^(Zhang et al., 2017)^, 52^(Hollands et al., 2020)^, 74^(Havens et al., 2014)^, 77^(Marlicz et al., 2012)^, 83^(Paczkowska et al., 2009)^, 94^(Jamiołkowska−Sztabkowska et al., 2022)^	11
Animal - Ovary	1^(Bhartiya, 2015)^, 31^(Ebrahim et al., 2021)^, 33^(Sriraman et al., 2015)^, 91^(Virant−Klun et al., 2016)^	4
Animal - Uterus	17^(Singh and Bhartiya, 2021)^, 26^(Singh et al., 2022)^, 78^(Gunjal et al., 2015)^, 88^(James et al., 2018)^	4
Animal - Testis	16^(Kaushik and Bhartiya, 2020)^, 20^(Kaushik et al., 2020)^, 41^(Bhartiya et al., 2012a)^, 98^(Anand et al., 2015)^	4
Human - Bone marrow (cadaver, critical limb ischemia patients)	49^(Delcroix et al., 2010)^, 59^(Guerin et al., 2015)^, 60^(Guerin et al., 2017)^	3
Human - Bone marrow	11^(D’Ippolito et al., 2006a)^, 79^(Garbayo et al., 2011)^, 28^(Rios et al., 2016)^, 85^(D’Ippolito et al., 2006b)^	4
Human - Testis	36^(Kurkure et al., 2015)^, 41^(Bhartiya et al., 2012a)^	2
Animal - Pancreas	62^(Bhartiya et al., 2014)^	1
Human - Wharton’s jelly/Placenta	66^(Iwasaki et al., 2009)^	1
Human - Heart	82^(El−Helw et al., 2020)^	1

Across the 98 studies examined, VSELs were successfully detected in 64 cases, and this represent approximately 65% of the total dataset. This has highlighted a consistent trend of positive identification across diverse experimental contexts demonstrated in Table 2.

**TABLE 2:** Summary of Experimental Findings Across 98 Studies on VSELs (Supplementary Table S1 - Article_Analysis). Related references are indicated in the Table 1.

Factors	Study IDs			
	Observed/Measured	Not observed/Not detected	Measurement/Detection not performed	Specific observation
Is VSELs detected?	1, 3, 4, 5, 7, 8, 12, 14, 15, 16, 17, 20, 21, 23, 24, 25, 26, 27, 31, 32, 33, 34, 35, 36, 38, 39, 40, 41, 43, 44, 45, 46, 47, 49, 50, 52, 54, 58, 59, 61, 63, 65, 66, 67, 68, 69, 70, 71, 72, 73, 74, 76, 77, 79, 81, 82, 86, 87, 90, 92, 93, 94, 95, 96, 97, 98	10, 11, 13, 18, 28, 42, 48, 51, 53, 55, 56, 57, 60, 62, 64, 89	2, 6, 9, 19, 22, 29, 30, 37, 75, 78, 80, 83, 84, 85, 88, 91	​
Do VSEL colonies “roll up” on the plate?	24, 44, 48	10, 11, 12, 13, 14, 15, 16, 17, 18, 30, 49	1, 2, 3, 4, 5, 6, 7, 8, 9, 19, 20, 21, 22, 23, 25, 26, 27, 28, 29, 31, 32, 34, 35, 36, 37, 38, 39, 40, 41, 42, 43, 46, 47, 50, 51, 52, 53, 54, 55, 56, 57, 58, 59, 60, 61, 62, 63, 64, 65, 66, 67, 68, 69, 70, 71, 72, 73, 74, 75, 76, 77, 78, 79, 80, 81, 82, 83, 84, 85, 86, 87, 88, 89, 90, 91, 92, 93, 94, 95, 96, 97, 98	33, 45 - small clusters formed
Do colonies form “spheroids” or lumps that float?	3, 24, 25, 45, 48, 64	8, 10, 11, 12, 13, 14, 15, 16, 17, 18, 19, 30, 44, 49	1, 2, 4, 5, 6, 7, 9, 20, 21, 22, 23, 26, 27, 28, 29, 31, 32, 34, 35, 36, 37, 38, 39, 40, 41, 42, 43, 46, 47, 50, 51, 52, 53, 54, 55, 56, 57, 58, 59, 60, 61, 62, 63, 65, 66, 67, 68, 69, 70, 71, 72, 73, 74, 75, 76, 77, 78, 79, 80, 81, 82, 83, 84, 85, 86, 87, 88, 89, 90, 91, 92, 93, 94, 95, 96, 97, 98	33 - clusters (“germ cell nests”) formed, floating oocyte-like structures in culture
Is teratoma formation observed in mice?	-	2, 3, 4, 5, 6, 7, 8, 9, 10, 11, 12, 13, 14, 15, 16, 17, 18, 19, 20, 21, 22, 23, 24, 25, 26, 27, 28, 30, 33, 35, 43, 44, 45, 47, 48, 49, 50, 51, 52, 53, 55, 59, 60, 61, 62, 63, 64, 65, 66, 67, 68, 69, 70, 71, 72, 73, 74, 75, 76, 77, 79, 80, 81, 82, 83, 84, 85, 86, 87, 89, 90, 92, 93, 94, 95	1, 29, 31, 32, 34, 36, 37, 38, 39, 40, 41, 42, 46, 54, 56, 57, 58, 78, 88, 91, 96, 97, 98	-
Have Pluripotency factors (NANOG, OCT4) and telomere length been studied?	1, 4, 5, 6, 8, 11, 12, 13, 14, 15, 16, 17, 20, 22, 24, 26, 27, 28, 29, 31, 33, 34, 35, 36, 38, 39, 40, 41, 43, 44, 45, 46, 47, 48, 49, 50, 53, 54, 62, 64, 67, 72, 73, 75, 76, 77, 78, 79, 81, 83, 84, 85, 87, 88, 90, 91, 95, 96	3, 7, 9, 10, 18, 19, 21, 23, 25, 30, 32, 37, 42, 51, 55, 56, 57, 58, 59, 60, 61, 63, 65, 66, 68, 69, 70, 71, 74, 80, 82, 86, 89, 92, 93, 94, 97	-	2 – only cell type characterised; 52, 98 - only a few markers are mentioned (e.g., OCT4 without NANOG) or the analysis is incomplete

Morphological hallmarks of VSEL colonies, such as “rolling up” behaviour on culture plates and spheroid or floating aggregate formation, were also reported in a subset of studies (3 and 6 studies respectively), with additional suggestive observations in others (e.g., small clusters or oocyte-like structures). Although teratoma formation which is often used as a functional test for pluripotency was not observed in any study, this may reflect either true biological properties or limited testing, as many studies did not perform such experiments. The issue of teratoma formation is often highlighted as a critical point (Cunningham et al., 2012; Lezmi and Benvenisty, 2022). Teratoma formation represents a classical hallmark of pluripotency and, simultaneously, a significant safety concern in advanced cell therapy. The presence of undifferentiated cells within a therapeutic product entails an inherent risk of uncontrolled growth and tumorigenesis, which regulatory agencies (EMA, FDA) and the ISSCR Guidelines (2021) explicitly require to be excluded before clinical application (Hyun et al., 2021; Lovell-Badge et al., 2021; Center for Biologics Evaluation and Research, 2024; Committee for Advanced Therapies CAT, 2025). Pluripotency indicates developmental potential, but at the same time underlines the necessity for rigorous safety validation.

In previous studies, no teratomas have been observed *in vivo* following autologous transplantation of adipose-derived stem cells in dogs under long-term monitoring (Bērziņš et al., 2018). The histological appearance of teratoma-like structures has been interpreted as evidence of broad differentiation capacity. Yet, such findings simultaneously emphasise the critical requirement to ensure the absence of undifferentiated cells before any translational use. Teratoma formation was detected only under hypoxic conditions during aggregate formation, whereas after expansion under normoxic conditions (21% O_2_) no teratomas were observed in BALB/c nude mice (Bērziņš et al., 2018).

These results are consistent with international recommendations emphasising the importance of long-term *in vivo* safety studies, particularly in large-animal models, as a strategy to mitigate tumorigenicity risks (Hyun et al., 2021; Lovell-Badge et al., 2021). The overall evidence indicates that pluripotency-associated risks are acknowledged, no teratomas have been observed *in vivo* under controlled conditions, and the available data support the translational potential of VSEL-like cells only when tumorigenicity is effectively managed.

Notably, pluripotency markers (NANOG, OCT4) and telomere length were assessed in 58 studies, strengthening the molecular characterisation of the VSEL phenotype. In several cases, multiple supportive lines of evidence such as marker expression and unique colony morphology coincided which reinforced the case for VSEL-like cell presence. Overall, despite methodological variation and occasional incomplete data, most studies offer converging evidence for the existence and unique biological features of VSELs, making this dataset a significant foundation for further validation and standardisation efforts.

### 4.8 Limitations of findings

This work did not aim to provide a full ISCT marker panel or quantitative marker proportions for the small-size fraction, nor does it present gating plots within the main text; instead, characterisation focused on telomere biology and stemness-associated features to support the heterogeneity hypothesis, and tri-lineage competence was confirmed on the total population prior to lineage-specific assays, consistent with ISCT minimal criteria and standard reporting guidance (Lee et al., 2008; Galipeau et al., 2016; Zhuang et al., 2021).

Future studies should implement a validated, indication-relevant potency assay matrix with predefined acceptance criteria anchored to the anticipated mechanism(s) of action (e.g., immunomodulation read-outs), as recommended by the ISCT potency framework (Galipeau et al., 2016). Differentiation assessments should be converted to quantitative read-outs—adipogenesis by Oil Red O imaging/quantification, osteogenesis by Alizarin Red S extraction or calcium assays, and chondrogenesis by sulphated glycosaminoglycan (sGAG) assays and histological scoring—to enable objective comparability across batches and sites (Gregory et al., 2004; Mehlem et al., 2013; Wang et al., 2018).

For hepatogenic differentiation, functional validation should include albumin secretion, urea production and cytochrome P450 activity as standard benchmarks for hepatocyte-like cells derived from MSCs (Aurich et al., 2009; Gilglioni et al., 2025).

Measurement assurance should be strengthened by reporting full cytometry metadata and gating in line with MIFlowCyt, and by standardising instruments with calibration beads and quality-assurance procedures to control inter-run variability (Lee et al., 2008; Perfetto et al., 2012).

Safety and function should be extended to adequately powered *in vivo* studies incorporating sensitive biodistribution methods, histopathology and longitudinal tumourigenicity assessment aligned with current points-to-consider (Reyes et al., 2017; Sato et al., 2019).

Release and stability testing should adopt good manufacturing practice (GMP)-aligned panels—including sterility, *mycoplasma* and endotoxin testing, cell viability, identity/purity, karyotype/genomic stability where appropriate, and shelf-life/traceability—in accordance with clinical-grade MSC manufacturing guidance (Sensebé et al., 2013; Lechanteur et al., 2021).

Finally, exploratory cell-state metrics (e.g., telomere length and NANOG/OCT4 expression) should be prospectively correlated with defined potency and safety endpoints, given their association with replicative senescence and risks of genomic instability during extended culture (Wang et al., 2013; Neri, 2019).

## 5 Conclusion

This novel experimental study charts the heterogeneity within human AD–MSCs populations, with a focus on the smallest cells and establishes their potential advantages in therapeutic applications. The overarching goal was to determine whether size-based sorting could isolate AD–MSCs with longer telomeres and therefore a higher regenerative potential.

During AD–MSC isolation, a method based on an early morphological indicator relying on the appearance of characteristic small colonies from 12 h to the first few days after initial seeding (P0) was developed. This method aided in isolating the smaller cell subpopulation, and only those cultures in which such colonies emerged were used for further processing. These were then compared to the unsorted population across several parameters. Key conclusions based on the fresh datasets and images collected can be described as:

- Distinct growth behaviour: The small-sized AD–MSCs exhibited unique growth characteristics. Unlike the standard flattened fibroblast-like monolayer, these cells spontaneously detached from the surface and rolled into large 3D spheroid-like aggregates within ∼96 h. This unusual behaviour suggests enhanced 3D organisation capacity and alters extracellular interactions, which appears to be potentially advantageous in next-generation tissue engineering.
- Longer telomeres: The smaller cells showed significantly long telomeres (∼18,000 bp) compared to the unsorted population (∼15,000 bp), indicating a younger replicative state. This supports the hypothesis that cell size correlates with biological age.
- Higher stemness marker expression: The small cell fraction expressed pluripotency markers Nanog and Oct4. This suggests that these cells may represent a more primitive MSC subpopulation, possibly resembling VSELs.
- *In vivo* safety and differentiation. In two parallel experiments in BALB/c nude mice (n = 3 per group), AD-MSCs that did not form floating aggregates produced no teratomas or teratoma-like structures (0/3). By contrast, in the case where cultures developed floating aggregates during the pre-implantation teratoma assay, AD–MSCs were injected together with their own aggregates—i.e., aggregates that had formed from the same AD–MSCs batch in the assay; and a single teratoma-like, well-vascularised lesion containing ectodermal elements (hair follicles) was observed in 1/3 mice, without malignant features. These data indicate that ectodermal differentiation can occur under conditions associated with floating-aggregate formation, whereas the absence of lesions in the non-aggregate arm suggests a condition-dependent risk. A pre-implantation safety workflow in which a teratoma assay is performed before clinical use enables prospective detection and exclusion of teratoma-forming AD–MSCs populations by selecting against cultures that develop floating aggregates. Given the small sample size and inconsistency of the signal, further studies are required to confirm the association, refine selection criteria, minimise teratoma-like lesion risk (e.g., by preventing aggregate formation or co-injection), and improve overall process controllability and safety.

In conclusion, cell size was found to provide a simple yet effective indicator for enriching MSC cultures with younger, potentially more potent cells. These findings open new possibilities for their use in regenerative medicine.

## Data Availability

The original contributions presented in the study are included in the article/Supplementary Material, further inquiries can be directed to the corresponding author.

## Supplementary material

SUPPLEMENTARY TABLE S1 List of primers used in the study - https://pmc.ncbi.nlm.nih.gov/articles/instance/4961112/bin/IJSC-09-124_suppl.pdf. Int J Stem Cells doi: 10.15283/ijsc.2016.9.1.124.
